# Military Hospitals in India

**Published:** 1917-02-17

**Authors:** 


### MILITARY HOSPITALS IN INDIA.

### By an Ex-Patient.

THE renewal of activity in the Mesopotamian campaign must, it is to be feared, again entail great activity in the above institutions. Again, too, we shall see, perhaps, newspaper reports of very grave charges made by ladies and others against their working or equipment. The present writer, who a dozen years ago worked in two or three Indian military hospitals, visited others, and in particular was a patient for a short time in one the name of which figured recently in the press to great disadvantage, would therefore like, as his testimony is of a very different import to that previously heard, to give his experience and impressions. These are necessarily a little out of date; but as the tendency in a growing study like medicine is everywhere forwards, and as nothing has happened in India during the last decade to oppose such a tendency in the particular case before us (indeed, rather the reverse), and as, on the top of this, the war must necessarily to some extent have strengthened medical claims, this experience of 1903-1904 cannot be considered irrelevant.

"EAST IS EAST AND WEST IS WEST."

In the first place, to get into the proper attitude towards this question the basic facts of the situation must be known and considered. India is a poor and primitive country, where the pay of a common labourer is sixpence a day. England is a rich and advanced one, where the labourer receives at least sixpence an hour. India is remote from European resources, England in close touch with them. What is possible in England is impossible in India, from material considerations alone. The authorities in the writer's time did not budget for an expenditure necessary to provide sick-beds in military hospitals with mosquito curtains to any extent, or for European nurses up-country, or for laboratory autoclaves better than made out of tin at the local bazaar—and quite well these answered for most purposes. But they did budget for paid Government entomologists travelling about to fight locusts and other enemies of the native farmers' crops. Again, India has a bad climate, England a healthy one. The enteric patient, debilitated for long previously with prickly heat and sleepless nights, lying in a high fever in a ward the air temperature of which kuss-kuss tatties will hardly keep below 100° F., will clearly not do so well on an average as the typhoid case at home. Yet again, medical arrangements in time of war must always in practice conform to military exigencies as interpreted by non-medical officials, and how ruthless of human life and limb and health such interpretation may have to be no one now needs telling. The unhampered arrangements of Guy's and St. Thomas's and the London are sometimes about as similar as a championship race in a swimming-bath is to a crew struggling in the water. Last, and least, as affecting amenities and appearances, the personal habits of Indians must be considered. Many sleep on the floor from choice; and although the writer's time abroad was too short to enable him to do a great amount of operative work, yet he never had any suppuration. A leg amputation and a radical cure of hernia healed *per primam* in that lowly situation just as well as the European soldiers' wounds did in bed.

It is a temptation to name the hospital already specially alluded to, but probably its officers would prefer obscurity. Suffice it to say that it is a large institution in a leading Indian city, well situated to catch the sea breeze which is that city's special alleviation. The writer went there seriously ill, left, in order to come home, a good deal improved, and is now quite recovered. He encountered there trained English nurses, cleanliness of surroundings as of a new pin, most kindly treatment, and even that great rarity in a land where the "mutton-club" is a necessary institution, tender meat. From the other officer patients, and they included the son of a renowned physiologist, he never heard the slightest complaint. It is possible, of course, that since those days its Commandant has been replaced by an incompetent sloven. But the explanation that somehow suggests itself is rather that the shrieking sisterhood, ignorant of war, ignorant of India, excited by the events of the time, so favourable to sensation and reaction, have been given facilities by a press which for years has been sinking in worth and dignity. There are Army medical reforms long overdue, it is true. One often felt furious that the same rough flannel shirt should be served out to the soldier in an Indian summer and in a Canadian winter. But the foregoing experience, and the position of high authority enjoyed by Indian Medical Service surgeons in, say, ophthalmology, has not left the writer in much doubt about the outcry already sufficiently alluded to.

